# A network analysis of psychological flexibility, coping, and stigma in dermatology patients

**DOI:** 10.3389/fmed.2023.1075672

**Published:** 2023-05-16

**Authors:** Vasilis S. Vasiliou, Hellen Russell, Sarah Cockayne, Gabriel Lins de Holanda Coelho, Andrew R. Thompson

**Affiliations:** ^1^School of Psychology, South Wales Clinical Psychology Doctorate, Cardiff and Vale University Health Board, Cardiff University, Cardiff, United Kingdom; ^2^Nuffield Department of Orthopaedics, Rheumatology and Musculoskeletal Sciences (NDORMS), University of Oxford, Oxford, United Kingdom; ^3^Department of Psychology, University of Sheffield, Sheffield, United Kingdom; ^4^Department of Dermatology, Sheffield Teaching Hospitals NHS Foundation Trust, Sheffield, United Kingdom; ^5^School of Applied Psychology, University College Cork, Cork, Ireland

**Keywords:** stigma, psychodermatology, process-based therapy, psychological flexibility, coping

## Abstract

**Introduction:**

Despite the negative effects of stigma in individuals with skin conditions, interventions to address its effects are rare. This might be in part due to a continued lack of understanding as to how individuals respond to stigma.

**Methods:**

In this study, we employed a step-case analytic method, using traditional regression, moderation, and network analyses, to examine the role of psychological flexibility (PF) with stigmatized experiences, and stigma-related outcomes. We run a cross-sectional study (*n* = 105 individuals with various skin conditions) and analyzed stigma-related variables. We included variables examining perceived stigmatization (PSQ), anxiety (GAD-7), depression (PHQ-9), well-being (EQ5D5L), and variables stemming from the PF model (CompACT), presented as three coping with stigma responses, namely “open,” “aware,” and “active.”.

**Results:**

Using network analysis, the most influential or central variables that contributed to stigma were generalized anxiety, perceived stigmatization, and valued actions. In relation to PF, being open to the experience of stigma (as opposed to avoidance), keeping a distance from stigmatized thoughts (as opposed to self-stigmatizing), and bringing attention to value-based committed actions (as opposed to passivity) were all found to contribute to less stigmatized experiences.

**Discussion:**

The results indicate that two of the three skills of the PF model (“open” and “active”) may be important targets for interventions targeting stigma in people living with skin conditions.

## 1. Introduction

Stigma is characterized by a proneness to either devaluate and discredit a person/group considered to possess a negative attribute (1), or an individual’s/group’s tendency to come to believe what others attribute to them (2, 3). Given the highly visible nature of skin conditions, it is unsurprising that stigma is commonly experienced (4–7). The visible marks on the skin can be perceived as “deviant” from what is considered the norm in appearance, making it easier for people in society to stigmatize individuals with skin conditions, compared to other conditions that show no visible differences in appearance (e.g., individuals with diabetes) (8, 9).

Existing research shows that stigma in various skin conditions, including acne, atopic dermatitis, vitiligo, and psoriasis, is associated with poorer quality of life and increased distress (7, 10–18). For example, individuals with psoriasis often feel “different” from others. This increases stigma-related stress, consequently, impacting individuals’ daily functioning (19). Further, studies in patients living with acne show that stigma is the largest contributor in predicting poorer quality of life, over and above disease and demographic variables (11). These findings are concerning, highlighting that individuals with skin conditions have to deal with the diagnosis/ management of the condition in addition to the potential negative effects of feeling stigmatized. Promoting approaches that focus on managing stigma and distress, is required, yet, this has proven to be difficult thus far to achieve (20, 21).

One approach that helps researchers and clinicians to identify effective responses to stigma is the process-based approach (22–25). This approach attempts to identify common responses to stigma that can be flexible enough so that they can concurrently target the contextual (e.g., stigmatization) and psychological (e.g., how individuals cope with thoughts and emotions) elements of stigma (20). A therapeutic approach that can target both the context of stigma and the way individuals respond to it is psychological flexibility (PF) (26–28). PF includes three trainable psychological skills, named “openness to experience” (defusion and acceptance), “behavioral awareness” (contacting the present moment and self-as-context), and “valued action” (values and committed action) that can be presented as “coping with stigma” responses. Research examining these PF-related skills on other conditions, such as stigma in relation to chronic pain or weight self stigma shows that the PF skills can buffer the effects of stigma (29–32). These sets of psychological skills are amenable to interventions (e.g., can be employed as coping with stigma responses outside of a therapy room) and can be delivered in different forms (e.g., digitally, in-group, one-to-one, etc.) (33–35). Yet, no research so far has examined how these skills can help individuals with skin conditions, experiencing stigma.

To date, the existing studies attempting to identify parameters of coping with stigma in this population are rare (6, 20). Further, existing studies have employed traditional methods to examine variables, such as mediation and moderation analyses that only present a static picture of how stigma, coping with stigma, and stigma-related outcomes interact. For example, McCleary-Gaddy and James (36) found mediating effects of stigma consciousness between skin tone, life satisfaction, and psychological distress among African Americans, highlighting the potential role of increased awareness of stigmatization in reducing distress. Further, Bohm et al. (37), and Schmid-Ott et al. (38) both found mediating effects of reduced self-esteem and rejection as stigmatization parameters in skin condition severity and quality of life, indicating the potential role of defusion from stigma related experiences as a coping response. Likewise, Krüger and Schallreuter (39) found behavioral avoidance as the main coping with stigma response in patients with vitiligo, and Lu et al. (40) found helplessness as an illness cognition response to stigmatization in patients with psoriasis and atopic dermatitis.

Overall, traditional moderation and mediation methods limit practical applications for intervention development targeting stigma (41, 42). This is because they may generate a wide range of skills (20), potentially increasing uncertainty about which skills to select and target (20, 43). Further, these approaches do not allow the dynamic and simultaneous bi-directional interaction of stigma-related thoughts, emotions, and behaviors (responses to stigma) to be studied. Given that stigma is a multi-dimensional construct (41, 42), new innovative data-driven methods that can address these complexities, such as network analyses, are needed.

Unlike traditional mediation and moderation analyses, network analysis explores relations between variables through partial correlations, which are visually illustrated with links (e.g., lines connecting different variables) that show the connection between the variables. Adopting such an approach would allow the conceptualization of stigma as a network of interactional patterns, centred around defining variables of interest, such as coping with stigma responses, and stigma-related outcomes, rather than artificially assigning variables into static dependent and independent variables (24).

A network analytic approach was taken in this study that tested the importance of variables and identified an empirically dynamic network of skills focusing on stigma alleviation. Stigma-related variables, including perceived stigmatization, anxiety, depression, well-being, and psychological variables, such as PF, were examined. In short, this study aimed to identify the most influential or central parameters contributing to stigma alleviation by attempting to determine (a) the relationships among all variables of interest, (b) the variance of stigma and PF skills in explaining individuals’ well-being; (c) the potential role of certain or all the three PF skills in buffering the effects of stigma; and (d) the bidirectional relations among the PF processes, stigma, and stigma related outcomes.

## 2. Methods

### 2.1. Design

The study was nested in a multi-center European study conducted by the European Society for Dermatology and Psychiatry (ESDaP)^1^. The ESDaP multi-country study collected data on the association between stigmatization and the psychosocial burden of individuals living with a skin condition in 17 European countries (ESDaP, 2016). In addition to the variables examined across all countries, some countries also investigated other variables. In the UK, the survey was expanded to include variables related to psychological flexibility so that the aims of this study could be addressed. The study had ethical approval from the NHS Health Research Authority (18/LO/0639).

### 2.2. Inclusion and exclusion criteria

Eligible participants were recruited from patients attending outpatient appointments with a dermatology department within a large teaching hospital in the UK. Inclusion criteria consisted of individuals over 18 years of age with a sufficient English capacity to complete questionnaires and provide consent, and a diagnosis of a chronic skin condition. The exclusion criteria consisted of a non-primary diagnosis of chronic skin conditions, the presence of a primary psychiatric condition relevant to skin distress (e.g., trichotillomania, delusional parasitosis etc.), a benign skin lesion (e.g., a noncancerous related skin lesion), and/or a suspected/diagnosed skin cancer.

### 2.3. Recruitment and study procedures

**Figure 1:**
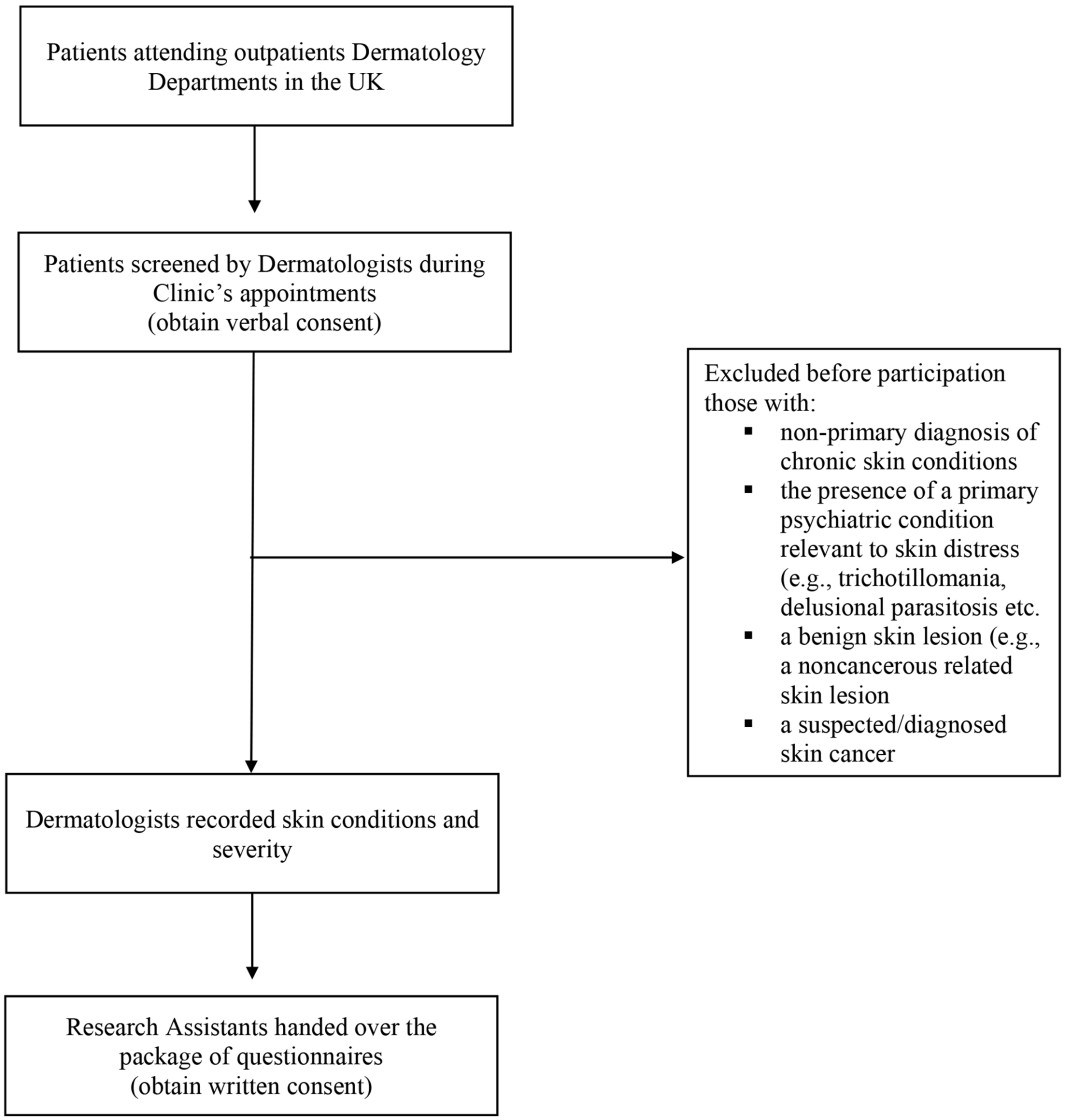
Flow chart with study procedures.

Eligible participants were recruited using convenience and purposive approaches. During clinic appointments, Dermatologists invited consecutive patients who met the study criteria to participate. Upon consent, participants completed the package of questionnaires with the assistance of a research team member, and study Dermatologists recorded their skin condition and severity. Dermatologists used the International Classification of Diseases (ICD-10) criteria to rate the participating individuals’ severity of their skin disease as mild, moderate or severe. Data collection occurred between July and September 2018. Figure 1 presents the flow chart with all the study procedures.

### 2.4. Measures

Participants completed a series of measures, including demographics, such as age, gender, education level, and employment status, clinically relevant questions about their skin conditions (disease severity and intensity), and a set of five standardized self-reported questionnaires, measuring stigma, depression, anxiety, quality of life, and a measure assessing the skills stemming from the PF, presented as three dyads or coping responses: “open,” “aware,” and “active.” In sum, the following measures were completed by the participants.

#### 2.4.1. Stigma-related variables

Perceived Stigmatization Questionnaire (PSQ) (44) consists of 21 items, assessing perceived stigmatization in social experiences (e.g., people avoid looking at me or people do not know how to act around me) in individuals with visible differences in appearance. Higher scores indicate a greater perception of stigmatized behaviors. The measure assesses stigmatized behaviors on a 5-point Likert scale (never, almost never, sometimes, often, always) and has good internal consistency and criterion validity with other related psychosocial constructs (e.g., good convergent and discriminant validity within a sample of adult burn survivors) (44). The Cronbach’s alpha for this study was a = 0.90.

#### 2.4.2. Psychosocial-related outcome variables

The Patient Health Questionnaire (PHQ-9) (45) is a self-administered questionnaire with 9 items, measuring the presence and severity of depressive symptoms (e.g., feeling down, depressed, hopeless or having little interest or pleasure in doing things). Participants are required to rate the frequency of nine symptoms of depression on a scale from “not at all (0)” to “nearly every day (3)” for the past 2 weeks. Total scores can range from 0 to 27. Depression is indicative of “mild” (scores 5–9), “moderate” (scores 10–14), “moderately severe” (scores 15–19), or “severe” depression (>20). The measure presents excellent internal reliability (Cronbach’s alpha = 0.89) and test–retest reliability (*r* = 0.84) (46), as well as an acceptance construct validity, as assessed by functional status (46). The Cronbach’s alpha for this study was a = 0.98.

The Generalized Anxiety Disorder Assessment (GAD-7) (47) is a self-administered 7 items measure of symptoms of a generalized anxiety disorder (GAD). The questionnaire asks participants to rate the frequency of nine symptoms of GAD within the last 2 weeks on a scale from “not at all” to “nearly every day” (scored 0–3 with a total score ranging from 0 to 21). Total scores can be interpreted of “minimal” (0–4), “mild” (5–9), “moderate” (10–14), or “severe” (15–21) anxiety. Research shows that the GAD-7 has excellent reliability (test–retest correlation of 0.83) and construct validity, as presented with correlations measuring functional impairment (47). The Cronbach’s alpha for this study was a = 0.98.

The EuroQOL 5-Dimensions (EQ5D5L) (48) is a visual analog scale (VAS) assessing self-reported health. Participants are asked to rate their health on the day of reporting (“today”), using a zero (“the worst health you can imagine”) to 100 (“the best health you can imagine”) metric. The validity of the EQ5D5L in skin populations shows good psychometric characteristics (49), showing moderate-to-strong correlations with other health-related quality-of-life measures (e.g., SF-12) and can detect significant changes in health status over time. The VAS is a subtest within this measure, and validation is not available for this subscale alone. Thus, the psychometric assessments refer to the whole EQ5D5L.

#### 2.4.3. PF related variables

The Comprehensive Assessment of Acceptance and Commitment Therapy Processes (*CompACT*) (50) is a 23-item measure of psychological flexibility with three subscales: openness to experience, behavioral awareness, and valued actions. The three factors represent latent constructs of PF skills, merged as dyads, reflecting acceptance and defusion (“open” being present; CompOE), present moment awareness and self-as-context (“aware” behavioral awareness; CompBA), and values and committed actions (“active”; doing what matters”; CompVA) (51). Participants respond to a series of items (e.g., I behave in line with my personal values) on a 7-point Likert scale from “strongly disagree” to “strongly agree.” Higher scores in each subscale or the total score indicate greater psychological flexibility (e.g., greater openness to experience, mindful attention to current activities and engagement in valued actions). The measure demonstrates excellent internal consistency in its subscales (*α* = 0.90, 0.87, and 0.90, respectively) and acceptable criterion validity with existing ACT measures, such as the Acceptance and Action Questionnaire (50). The Cronbach’s alpha for this study was for the CompOE *a* = 0.88, for the CompBA *a* = 0.91, and for the CompVA *a* = 0.93, correspondingly.

### 2.5. Statistical analyses

As part of the preliminary analysis, we examined the parametric assumptions and tested the normality of distribution by visually inspecting the histograms, P–P residual plots, and missing cases. We did not detect a serious violation of the normality assumptions (linearity, homoscedasticity, collinearity, and multicollinearity). Also, univariate and multivariate outliers and missing cases were negligible. We examined the histograms and plots for any issues with the skewness and kurtosis. There were no values below or above the −/+3. The measures of psychological distress (anxiety and depression) were positively skewed. Ratings of self-reported health were negatively skewed. There was a notable outlier in the stigma data, with one participant scoring very high on the Perceived Stigmatisation Questionnaire (49, *z* = 3.15). We rerun all the reported analyses without the outlier and conclusions drawn were the same. Therefore, the reported results include the outlier as it was deemed a genuine, although extreme score. Hence we left the data intact. To corroborate with the visual inspection of the dataset, we run a missing data analysis to assess any pattern of non-identifiable missingness (52). Little’s MCAR test indicated that the data were missing completely at random.

The main analytic plan followed an exploratory step-wise approach. We first explored a “static” or pre-defined model of variables, using traditional regression and moderation regression analyses, to examine predictive relationships among the variables (e.g., stigmatized experiences as predictors of distress and low perceived health). We then examined “dynamic” and “bidirectional” relationships of the variables using network analyses. We used IBM SPSS Statistics 27 to test and compare the variables’ importance. We then used the packages of JASP^2^ and R studio (53) to run the network analysis.

Firstly, we run a series of Pearson’s correlation coefficient analyses (54) and a series of hierarchical multiple linear regression analyses (simultaneous forced entry method using *R*^2^ and adjusted *R*^2^), to examine the prediction of stigma on well-being, controlling for any effects of age, gender, and clinician-rated severity of the skin condition. Then, we performed a series of moderation analyses, to test whether PF moderated the relationship between feelings of stigma and well-being. We then run a network analysis to simulate a hypothesized stigma model and identify the most central, therefore, most influential, PF skills that correlated with stigmatization and stigma-related outcomes. We examined the partial correlations network (total scale/subscale scores, rather than individual items) of the PSQ (stigma), GAD (generalized anxiety), PHQ9 (depression), EQ5D5L (perceived health), and the three sub-scales of the *CompACT*, CompOE (open to experience), CompVA (values), and CompBA (Behavioral awareness). Using the glasso R package (55) embedded in the JASP, we depicted graphically the edge weights connecting the nodes (e.g., the variables included in the model) and examined the nodes’ strengths. We also used the Fruchterman-Reingold positioning algorithm (56)- a forced-directed method- to visualize the network model variables and examine which variables are posed in the center of the graph.

For the interpretation of the outcomes, we applied the graphic LASSO [Least Absolute Shrinkage and Selection Operator (57)] estimator [a stunning parameter set to 0.5-using the EBIC; Extended Bayesian Information Criterium (58)], to counterbalance the relevant small sample size of the study (model regularization) (59). The technique estimates the variance–covariance matrix and removes less relevant edges from the model, returning a parsimonious network of partial correlation coefficient which is more conservative and easily interpretable (e.g., only a reasonably small number of edges are used to explain the covariation structure of the model). We also examined the stability of centrality indices using a parametrization technique called *bootent* (59) in the *R* software (53). We estimated the Coefficient Intervals (CIs), to examine if the order of centrality indices remains the same after bootstrapping (re-estimating) the network with fewer cases (e.g., dropping cases from the original dataset) and without replacing them. To assess this stability, we used the correlation stability coefficient, or CS-coefficient (quantification of stability). CS-coefficient defines the percentage of cases that can be dropped, with a 95% probability of maintaining ~0.70 correlation, compared to the completed data (59). The edge-weight accuracy is estimated when values are over 0.50 but not lower than 0.25. Finally, we examined the edge weights CIs to assess the precision with which PF processes are strongly interconnected within the network. Narrower CIs indicate better accuracy (59).

### 2.6. Statistical power and sample size

The proposed analysis included a maximum of seven variables to detect medium effect sizes in the first round of analyses which included multiple regression and moderation analyses. Following suggestions from Cohen and Field (60, 61) a G* power analysis (62) suggested a sample size of 105 participants, for *p* < 0.05. For the second round of network analysis, the number of observations in our tested model (e.g., *n* ~ 100) seemed appropriate for estimating the partial correlation network analysis. That is, we expect 20 nodes to occur on the network model, allowing us to examine the validation and robustness of the model even when the highly conservative Lasso penalty estimator is applied (59).

## 3. Results

### 3.1. Sample and descriptive characteristics

One hundred five participants filled out the questionnaires, and 57% (*n* = 59) were women, with a mean age of 54 (ranging from 19 to 90). Most of the participants had completed the highest level of education (GCSE equivalent or below; 63%, *n* = 66), with more females (*n* = 46) than males (*n* = 20), achieving the highest level of education. Most of the participants were, at the time of the study, employed (41%, *n* = 43) or retired (40%, *n* = 42). As for the participants clinical characteristics, among the 29 reported primary skin conditions, the most common diagnoses were: psoriasis (*n* = 23), eczema (*n* = 16), and alopecia (*n* = 11). Other skin diseases diagnoses that occurred in >3% of the sample, included acne (*n* = 7), rosacea (*n* = 3), and urticaria (*n* = 2). Skin disease diagnoses given in >1% are presented in the Supplementary material S4 where we also present the comprehensive list of participants’ skin diagnoses. Clinicians’ ratings of the severity of participants’ skin disease were most commonly moderate (45%, *n* = 44) or severe (34%, *n* = 36). There were no differences between males and females in the employment status and clinicians’ rated severity of skin diseases (both *ps* < 0.05). Table 1 presents more detailed characteristics of participants’ demographic information.

**Table 1:** Participants characteristics.

Characteristic^1^	Sex			Total
	Male (*n* = 44) (Mean, *N* or %)	Female (*n* = 59)	*T* r *x*^2^ (*p/df*)* *n* = 105	*n* = 105 (Mean, *N* or %)
Age	52.50 (16.96)	54.88 (18.71)	0.26	53.86 (17.94)
Educational level (% years completed)			0.002 (*2*)	
GCSE or below	20	46		62.9% (*n* = 66)
A Level or equivalent	7	3		9.5% (*n* = 10)
Degree or above	18	11		27.6% (*n* = 29)
Employment Status			0.11 (*5*)	
Unemployed	6	9		14.3% (*n* = 15)
Retired	13	29		40% (*n* = 42)
Sick leave	1	0		1% (*n* = 1)
In education	1	1		1.9% (*n* = 2)
Employed	24	19		41% (*n* = 43)
Clinician rated severity of skin disease			0.21 (*3*)	
Mild	7	13		19% (*n* = 20)
Moderate	15	32		44.8% (*n* = 47)
Severe	21	15		34.3% (*n* = 36)
Descriptive Characteristics^2^				
Stigma (PSQ score range 0–27)	16.64 (*11.18*)	12.61 (*10.68*)	0.99 (*100*)	14.42 (*11.04*)
Anxiety (GAD 7 score range: 0–21)	6.31 (*6.04*)	6.95 (*6.23*)	0.37 (*100*)	6.67 (*6.12*)
Depression (PHQ 9 score range: 5–27)	7.43 (*6.96*)	8.03 (*7.85*)	0.20 (*100*)	7.77 (*7.45*)
Self-rated health (EQ5D5L score range, 0–100)	67.45 (*17.73*)	66.04 (*22.92*)	0.06 (*99*)	66.65 (*20.73)*
CompOE (Open; open to experience)	31.02 (*9.40*)	30.75 (*9.89*)	0.53 (*97*)	30.87 (*9.63*)
CompBA (Aware; Behavioral awareness)	16.64 (*6.49*)	17.04 (*7.49*)	0.22 (*97*)	16.86 (*7.03*)
CompVA (Active; Doing what matters)	35.11 (*9.16*)	34.38 (*9.95*)	0.61 (*97*)	34.71 (*9.57*)

1 Mean comparisons between groups were executed with independent *t*-tests for continuous variables and ×2 fisher tests for categorical variables. Due to missing, the overall sum up does not equate *n* = 105 in all variables examined.

2 Descriptive characteristics present means and standard deviation of the total scores for the study variables, split into males and females.

### 3.2. Correlation analyses

The stigma experience scale (PSQ) score demonstrated medium negative correlations with the openness to experience subscale score (CompACT_OE, *r* > −0.33), the behavioral awareness (CompACT_BA, *r* > −0.27) scores of the PF processes, and the perceived health (VAS, *r* > −0.24) scores. Further, stigma showed a positive correlation with the study outcomes, such as higher levels of stigma experiences being associated with higher levels of depression (PHQ-9, *r* > 0.34) and generalized anxiety (GAD-7; *r* > 0.29). As Table 2 shows, these findings support the first study hypothesis, indicating a significant relationship between stigma, PF processes, and stigma-related outcomes, consequently, allowing us to build the predictive models.

**Table 2:** Correlations between predictor variables (stigma), mediators (PF processes), and outcome variables (stigma-related impact).

	1	2	3	4	5	6	7
1. PSQ		−0.33**	−0.27**	−0.15	0.34**	0.29**	−0.24*
2. CompACT_OE	−0.33**		0.68**	0.08	0.58**	−0.65**	0.34**
3. CompACT_BA	−0.27**	0.68**		0.22	−0.58**	−0.63**	0.41**
4. CompACT_VA	−0.15	0.08	0.02		−0.23*	−0.28**	0.30**
5. PHQ-9	0.34**	−0.58**	−0.58**	−0.23*		0.84**	−0.62**
6. GAD-7	0.29**	−0.64**	−0.63**	−0.28**	0.84**		−0.61**
7. VAS	−0.24*	0.34**	0.41**	0.30**	−0.62**	−61**	

All correlations are Pearson’s r; *n* = 107; PSQ, perceived stigmatization questionnaire; CompACT_OE, openness to experience; CompACT_BA, behavioral awareness; CompACT_VA, valued actions; PHQ-9, patient health questionnaire; GAD-7, generalised anxiety disorder; VAS, EQ5D5L.

** *p* < 0.01.

* *p* < 0.05.

### 3.3. Multivariate analyses

The hierarchical multiple regression models consisted of seven predictors. We firstly entered (forced entry) demographics and clinical characteristics (step 1), followed by stigma (step 2), and finally, the three PF dyads of response processes (step 3). Before running the models, we log-transformed anxiety, depression, and self-reported health variables as they did not meet the criteria for normality due to skewness. Screening criteria showed no multicollinearity or the presence of multivariate outliers, and the variables met the criteria for normality, linearity, and homoscedasticity. For all the models, the variance inflation factor (VIF) was less than 3.3, and tolerance statistics were all 0.296 or above.

As Table 3 shows, the seven predictors, after controlling for demographics and clinical characteristics accounted for 57% of the variance explained in generalized anxiety (adj. *R*^2^ = 0.53). The equation was highly significant [*F* (7,95) = 16.53, *p* < 0.001], representing a large effect size, *f*^2^ = 1.14. Age, skin condition severity, stigma, and the three PF response styles were all significant predictors in the final model, with behavioral awareness (CompACT_BA) showing the highest contribution (*b* = −0.451) when compared with the other six predictors. In predicting depression, the seven predictors accounted for 38% of the variance (Adj. *R^2^* = 0.379). The equation was highly significant [*F* (7,95) = 9.28, *p* < 0.001], representing a large effect size *f*^2^ = 0.85. Examining the individual prediction (criterion) of the seven variables, one can see that stigma and valued-based actions approached significance (*p* = 0.07). In contrast, the two other PF dyads, openness to experience and behavioral awareness were significant. The variable with the highest prediction was behavioral awareness (*b* = −0.34, *p* < 0.01) compared to the other six variables. Finally, as for the perceived health, the overall model accounted for 22% of the variance explained (Adj. *R^2^* = 0.218). This finding was also highly significant [*F* (7,95) = 1.69, *p* < 0.01], representing a large effect size *f*^2^ = 0.52. Behavioral awareness and value-based actions were the only significant predictors in the final model, with an almost equal prediction of perceived health (*b* = −0.281 and *b* = −0.241). The regression analyses supported the second study hypotheses, where perceived stigmatization predicts higher anxiety, depression, and lower self-reported health. Notably, PF processes might revert the negative effects of stigma on individuals’ well-being, particularly the process of behavioral awareness (being present). We tested which PF processes of change exert effects in the following analyses.

**Table 3:** Linear regression for the prediction of anxiety, depression, and perceived health.

Independent variables (Predictors)	Steps (blocks)^1^	*Β**^2^*	*t*	*p*	*R^2^*	*Adj. R^2^*	*F (Df)*	*p*
Dependent variable: anxiety
Age	1	−0.151	−2.210	0.030	0.051	0.020	1.66 (3,95)	0.18
Gender		0.036	0.417	0.677				
Severity		−0.024	−0.024	−0.024				
Stigma (PSQ)	2	0.005	2.48	0.015	0.11	0.072	2.85 (4,95)	0.03*
Openness to experience (CompACT_OE)	3	−300	−3.07	0.003	0.57	0.534	16.53 (7,95)	<0.001
Behaviorsal Awareness (CompACT_BA)		−0.451	−4.71	<0.001				
Valued-based actions (CompACT_VA		−0.180	−2.52	0.013				
Dependent variable: depression
Age	1	0.027	0.326	0.745	0.004	−0.028	0.13 (3,95)	0.93
Gender		0.018	0.209	0.835				
Severity		−0.012	−0.148	0.883				
Stigma (PSQ)	2	0.196	1.885	0.063	0.132	0.094	3.46 (4,95)	0.011
Openness to experience (CompACT_OE)	3	−0.258	−2.285	0.025	0.425	0.379	9.28 (7,95)	<0.001
Behaviorsal Awareness (CompACT_BA)		−0.343	−3.107	0.003				
Valued-based actions (CompACT_VA)		−0.149	−1.816	0.073				
Dependent variable: perceived health
Age	1	0.085	0.881	0.381	0.013	−0.019	0.41 (3,95)	0.742
Gender		0.021	0.213	0.832				
Severity		0.105	1.075	0.285				
Stigma (PSQ)	2	0.140	1.338	0.184	0.079	0.038	1.95 (4,95)	0.109
Openness to experience (CompACT_OE)	3	−0.032	−0.243	0.808	0.218	0.156	1.69 (7,95)	0.002
Behaviorsal Awareness (CompACT_BA)		−0.281	−2.185	0.032				
Valued-based actions (CompACT_VA		−0.241	−2.520	0.014				

1 Variables were entered simultaneously in blocks (steps) and each independent variable was assessed in terms of what it adds to the prediction of the dependent, when the previous variables were controlled for.

2 Beta represents standardized coefficients to the equation to allow for comparisons.

### 3.4. Moderation mediation analysis

We conducted a moderated regression analysis to assess whether PF (total score on the CompACT questionnaire) moderates the relationship between stigma and well-being. We hypothesized that higher levels of PF would indirectly buffer the negative effects of stigma and stigma-related outcomes. To test for moderation, stigma, PF, and their interaction was entered together in a single block to three models, predicting generalized anxiety, depression, and perceived health. Variables were mean-centered prior to computing the interaction terms to minimize multicollinearity problems. A significant interaction term would indicate the presence of moderating effects.

As Supplementary material S1 shows, none of the moderation analyses were significant. For example, when stigma and PF were entered together, they explained 50% of the variance in log anxiety *R^2^* = 0.50, *F* (3, 91) = 28.92, *p* < 0.001, but the interaction term was not a significant predictor of anxiety. For depression, when the same variables were entered together (stigma and PF), they explained 38% of variance in log depression scores *R^2^ = 0*.38, *F* (3,93) = 20.63, *p* < 0.001, but again the interaction was not significant. Finally, the same results were observed for perceived health where stigma and PF explained 16% of the variance in log self-reported health *R^2^* = 0.16, *F* (3,94) = 7.2, *p* < 0.001, yet the interaction was not significant. In sum, the third aim was not supported, indicating that the relationship between stigma, PF processes, and stigma-related outcomes appears to be more complex and dynamic than static, as these predictive models indicate. To examine the dynamic role of the PF processes, we finally run a network analysis.

### 3.5. Network analysis

**Figure 2:**
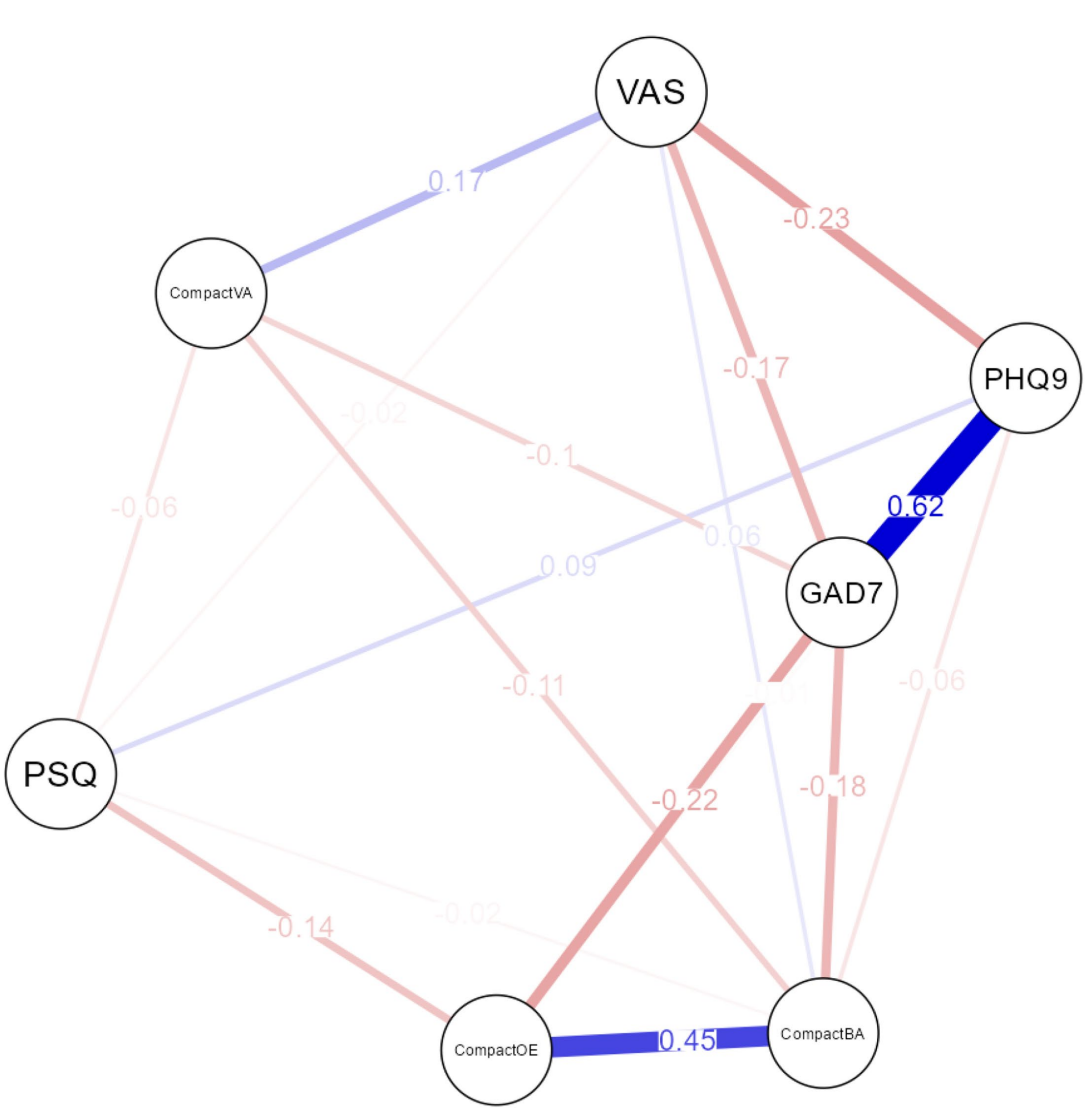
Network model of Stigma, PF responses skills (dyads), and stigma-related outcomes. Red edges indicate negative partial correlations; blue edges indicate positive partial correlations; PSQ: Perceived Stigmatisation Questionnaire; PHQ9: Patient Health Questionnaire; GAD7: Generalised Anxiety Disorder Assessment; VAS: EuroQOL 5- Dimensions- EQ5D5L Visual Analogue Scale; CompactOE: openness to experience; CompactBA: behavioral awareness; CompactVA; valued actions.

The final network is illustrated in Figure 2. Based on the strength centrality indices, the node with the highest centrality, and therefore the most influential within the model, was generalized anxiety (GAD-7), followed by perceived stigmatization (PSQ), valued actions (CompACT_VA), and depression (PHQ-9). As expected, the model’s strongest (more meaningful) positive relations, excluding the PF, were observed between depression and anxiety, and stigma and depression (see Supplementary Table S2 for all the variables examined weights partial correlations). The strongest negative relationships were observed between anxiety and perceived health, and depression and perceived health.

We found strong positive relationships between open and aware, and active and perceived health. The strongest negative relationships of PF with stigma were observed between open and anxiety, aware and anxiety, and open and stigma. Table 4 presents the edge weights partial network correlations of PF processes when LASSO regularization was applied. Stigma had the strongest negative relationships (edge) with openness to experience (weight matrix), followed by valued actions and behavioral awareness. Further, generalized anxiety was also found to exert a large negative relationship with openness to experience, followed by behavioral awareness, and valued actions. Depression was only found to be negatively related to behavioral awareness and positively to stigma. The Supplementary Table S2 presents all the relevant partial correlations among the examined variables. When percentages of cases were dropped off, stability assessment showed that the order of node strength was interpretable with some care. The edge weight accuracy (CIs) was found narrow for most PF processes when interconnecting with other nodes (see Supplementary material S3).

**Table 4:** LASSO regularized partial correlation coefficients for PF processes.

Psychological flexibility processes	Stigma	Emotional functioning		Daily functioning
		Anxiety	Depression	Perceived healthy (QoL)
Openness (Compact OE)	−0.142	−0.218	−0.010	0.000
Awareness (Compact BA)	−0.022	−0.177	−0.060	0.170
Active (Compact VA)	−0.062	−0.102	0.000	0.055

LASSO estimator was applied to controls for spurious (non-reliable) relations and to return a sparse (conservative) network model where only a relatively small number of edges are used to explain the covariation structure in the data. Therefore, edge (nodes) relations estimated as 0.00 reflect negligible relations within the model.

## 4. Discussion

Stigmatization is a common problem associated with living with a skin condition, yet relatively little is known about how this is influenced by psychological variables associated with distress. Network analysis has the potential to examine the multifaceted and bidirectional interactions associated with several variables potentially relevant to stigmatization in skin conditions (1, 59). In this study we specifically examined the relationship between psychological flexibility (PF), quality of life, stigmatization, and distress.

Findings showed that stigma was negatively related to the three skills PF associated with depression and anxiety. Behavioral awareness accounted for the largest portion of variance explained among the three skills of PF response styles (open, aware, and active), predicting lower anxiety, depression, and higher perceived health. Moderation analyses showed no effect of the three PF response styles between stigma and outcomes. This finding suggests that PF responses may not be considered as static-not amenable to direct change variables, but as dynamic, sharing some potentially therapeutic role in buffering the effects of stigma in individuals with skin conditions. To further examine our hypothesis, we run a network analysis. Findings indicated generalized anxiety, depression, perceived stigmatization, and value-based actions as the most highly interconnected variables within the network. Stigma was most strongly negatively associated with avoidance (as opposed to being open) and value-based actions (as opposed to being active), and positively with anxiety and depression. These findings are congruent with existing research demonstrating the negative role of stigma in increasing psychological distress to individuals with skin conditions. However, our study provides support for the role of the PF responses as trainable skills that may play central role in tackling stigma. As such, these responses may be foci for interventions, designed that can lower stigma-related distress.

The role of depression and anxiety is consistent with studies on stigma (7, 63, 64). In our study, we observed depression as the only variable associated with stigma in the network model. Concerning stigma, depression in individuals with skin conditions might be seen as a form of avoidance and passivity behaviors (39). These behaviors can lead individuals with skin conditions to avoid seeking support as a result of of stigma and shame (65). On the other hand, anxiety can be seen as a form of social anxiety related to the visible difference in appearance, further supporting some studies, showing that social anxiety is the most common form of distress for this population (66, 67). In this case, a measure assessing generalized anxiety disorder (GAD-7) might not entirely capture the distress individuals with skin problems experience.

The findings in this study point towards the role of PF skills as effective responses to stigmatization. More specifically, findings from the network analysis indicated the influential central role of the “*open*” response style (comprising the PF skills of acceptance and defusion) as a promising intervention target, to reduce the effects of stigmatized behaviors in people with skin conditions. Existing research shows that being willing to experience both internalized stigma (e.g., when individuals come to believe the stigmatized thoughts) and enacted stigma (e.g., when others impose stigmatized attitudes) can reduce stigma-related distress and improves daily functioning (34, 51, 68). In our case, such finding suggests that being more open and engaged allows individuals with skin conditions first to acknowledge more willingly that their visible difference in appearance may trigger stigmatized reactions and correspondingly respond to stigma more openly by minimizing avoidance (e.g., attempting to control one’s stigmatized behaviors) and by abstaining from attaching rigidly to stigmatized thoughts (e.g., seen stigmatized thoughts as true literal entities that can define behaviors) (29, 30, 69). Findings from research in the area of social psychology resonate with this approach of awareness vs. control of stigmatized behaviors (70, 71) or changing self-stigmatized thoughts (72), yet further research is warranted, especially, to indicate how being open to stigmatized experiences, is an effective practice for individuals with skin problems.

Additionally, the study showed the important role of the “*active*” response style (comprising of the PF skills of values and committed actions), indicating foci for intervention development. Findings showed value-based actions as one of the most influential nodes in the network, exerting a negative association with stigma, anxiety, and a positive one with perceived health. Other studies indicates that values can lower distress and increase daily functioning (73–75). Value-based interventions help individuals identify and clarify their values, shift attention toward value-based actions in moments of distress, and guide them to resonate with those choices (76). Because stigma can promote a disconnect and a dissonance from one’s values (77, 78), the process of increased attention to value-based actions may not directly impact the cognitive or emotional content related to stigma but cultivate engagement of individuals’ to more healthy behaviors, such as adhering to medical prescription or taking care of ongoing flares due to the disease’s progress (79). Consequently, this can increase the frequency where healthy behaviors are chosen in different contexts where stigma occurs (e.g., “*I can see others frown their eyes when they spot my pale white patches on my face, but this does not stop me from enjoying shopping in the mall or attending a social event”).* Research indicates values as the process that increases motivation towards health behavioral changes (31, 80), yet, future research will shed more light on how individuals with skin conditions, in particular, can use values in this way, even in the presence of stigma.

The present study findings are noteworthy, suggesting both theoretical and clinical implications. From a theoretical point of view, our findings indicate two of the three PF response of psychological flexibility as being essential to tackle the effects of stigma and related psychological distress. This contains a set of trainable behavioral responses that allow individuals to address concurrently core psychological, behavioral, and contextual parameters of perceived stress (27, 81), such as stigma. Because these skills reflect common responses to perceived threats (e.g., stigmatized behaviors), we can more directly specify what are the core functionally important pathways that we can focus on and change. Theoretically, for this to occur, we first need to link how individuals respond to stigma. Findings from this study indicated the use of the “*open*” and “*active*” response styles of the PF as skills that hold the potential to reduce the effects of stigma. Secondly, we need to find approaches that incorporate all the relevant past, present and contextual factors (e.g., demographic, disease severity, health care professionals’ behaviors) that seem to contribute to the psychological reaction involved in stigma (e.g., social anxiety and avoidance). Notably, we can achieve this level of analysis by employing methods, such as momentary ecological assessments that can collect high temporal personalized density data at the context of individuals’ lives (82). As a first step towards this approach, our findings indicated foundational knowledge about the nuances of unidirectional and bidirectional relationships of stigma-related associations within a nomological network that goes beyond static correlational, regression, and moderation analyses. Such a level of analysis can propose future directions and indicates clinical progress (83).

From a clinical perspective, focusing on functionally important skills, clinicians can develop scalable interventions for stigma that can meet the needs of a heterogeneous group of individuals with skin conditions (21). For example, the open response style should be employed when the problem is a narrow response to self-stigmatization where individuals attempt to reduce the stigmatized thoughts or replace them with more neutral or informative ones. On the contrary, when individuals respond to stigma with avoidance or passivity, values and commitment to health behaviors (as opposed to avoidance) should be employed. As stigma is a multidimensional phenomenon, focusing solely on individuals’ responses as the main intervention to tackle the effects of stigma, is likely to be suboptimal. One should move beyond skills and attempt to understand stigma as a context-specific problem, including biophysiological and sociocultural levels of stigma. Consistent with the network intervention approach, these skills should not be seen as snapshots that can be delivered across skin conditions. Rather, they ought to be seen as dynamic and interconnected systems of an intervention that are likely to modify person-specific coping with stigma responses, including broader sociocultural parameters that feed into the stigma. This requires a deliberate shift to models that organize different intervention strategies into a more coherent network (25). Such a model is the new Extended Evolutionary Meta-Model [see further here (81)]. It is based on evolutionary science and allows interventions to expand targeted PF skills, including conceptions about adaptation and resilience (84–86).

The present study had several limitations. First, the study used self-reported subjective measures known for their source bias and shared method variance. Secondly, the study was part of a larger cross-sectional epidemiological study that employed only a few psychosocial parameters involved in stigma. While we present new knowledge using variables that indicated the “central” role of PF, other variables that were not included, should be measured for a more integrated interpretation of stigma, such as contextual, interpersonal, and functional (6). Likewise, we made use of the UK-only self-selected sample, and this narrows the interpretation of the findings to predominantly white Caucasian populations. Equally, the sub-sample that measured stigma and PF parameters was underpowered for network analysis. Although the network model stability was found to be within acceptable ranges, interpretation should be cautious as the interpretation of CIs in analyses such as LASSO regularization is problematic because the initial estimates of network analyses are biased towards zero (59). Therefore, further replication of the study findings is warranted.

Future research should attempt to collect multiple and large-scale data, using measures that examine the experience of stigma holistically, with samples from different countries and with more heterogeneous skin conditions. This will allow researchers to use network comparison analyses and explore coping with stigma-related outcomes interconnections, including several contextual characteristics (e.g., demographics, race/ethnicity, disease onset or progress, etc.). Further, as the affective component of body image (e.g., anxiety, distress, shame, etc.) may be related to specific aspects of physical appearance (21), future studies should use disease-specific measures to assess affection. Likewise, future studies should attempt to examine stigma and coping responses, employing more idiographic and personalized methods, such as ecological momentary assessments (EMA). These methods can longitudinally collect behavioral and self-reported highly temporal data to assess the impact of targeted skills on stigma in the context of within-person variability, indicating personalized interventions.

This study applied step-wise analytic approaches to individuals with skin problems. Among the examined variables, stigma, depression, and two of the three response styles of the PF model, namely “*open*” and “*active*” skills, appeared important. The role of PF in the network analyses indicate certain functionally important pathways that may have clinical utility in psychosocial programs, attempting to reduce the effects of stigma in skin populations. Tailoring personalized approaches may increase the likelihood of a truly good outcome for individuals with skin problems, experiencing stigma. For this to occur, researchers and implementation scientists should employ newest approaches, such as the process-based intervention approach (25) and the Extended Evolutionary Meta-Model (EEMM) (81) as guides to develop a coherent network of intervention strategies that will tap across the multiple nature of stigma.

## Data availability statement

The data analyzed in this study is subject to the following licenses/restrictions: data are part of a larger European epidemiological research and can be available upon request. Requests to access these datasets should be directed to AT, thompsona18@cardiff.ac.uk.

## Ethics statement

The studies involving human participants were reviewed and approved by NHS Health Research Authority (18/LO/0639). The patients/participants provided their written informed consent to participate in this study.

## Author contributions

VV: writing – original draft (lead), methodology (equal), formal analyses (equal), and writing – review & editing (equal). HR: conceptualization (equal), formal analysis (equal), project administration (lead), and resources (equal). SC: investigation (lead) and resources (equal) GC: software (lead). AT: methodology (equal), conceptualization (equal), writing – review & editing (equal), and supervision (lead). All authors contributed to the article and approved the submitted version.

## Funding

This manuscript was prepared as part of the first co-author Post-doctoral Research Associate Position in Clinical Health Psychology which is funded by the Health Education and Improvement Wales (HEIW). The data collection was fulfilled as part of the partial completion of the second co-author's doctoral in Clinical Psychology program (D.Clin.Psy) which was conducted at the University of Sheffield and funded by the Health Education England, United Kingdom.

## Conflict of interest

The authors declare that the research was conducted in the absence of any commercial or financial relationships that could be construed as a potential conflict of interest.

## Publisher’s note

All claims expressed in this article are solely those of the authors and do not necessarily represent those of their affiliated organizations, or those of the publisher, the editors and the reviewers. Any product that may be evaluated in this article, or claim that may be made by its manufacturer, is not guaranteed or endorsed by the publisher.

## Footnotes

^1^ https://www.psychodermatology.net/

^2^ https://jasp-stats.org/
