# Supplementary material for: A network analysis of psychological flexibility, coping, and stigma in dermatology patients
Source: Front Med (Lausanne). 2023 May 16;10:1075672. doi: 10.3389/fmed.2023.1075672 (PMC10227518; doi:10.3389/fmed.2023.1075672)
Supplement: Supplementary file 1 [file Data_Sheet_1.docx]

Supplementary Material

**Supplementary Table S1:** Results of Moderation Analysis for Anxiety, Depression, and Perceived Health

| Variable | *B* | *SE B* | β | *95% CI* Lower | *95% CI* Upper |
| --- | --- | --- | --- | --- | --- |
| Generalized Anxiety | | | | | |
| Stigma | .0005 | .007 | .006 | -.013 | .014 |
| PF | -.034 | .004 | -.68*** | -.042 | -.027 |
| Stigma x PF | .00007 | .0004 | .017 | -.001 | .001 |
| Depression | | | | | |
| Stigma | .018 | .008 | .20* | .002 | .035 |
| PF | -.030 | .005 | -.55*** | -.039 | -.021 |
| Stigma x PF | .00006 | .0004 | .011 | -.001 | .001 |
| Perceived Health | | | | | |
| Stigma | .010 | .007 | .15 | -.004 | .024 |
| PF | -.015 | .004 | 1.38*** | -.023 | -.007 |
| Stigma x PF | .0003 | .0003 | .079 | -.0004 | .001 |

Note: * Significant at *p* < .05, *** significant at *p* < .001.

**Supplementary Table S2:** LASSO Regularized Partial Correlation Coefficients for all variables examined

|  | Network Weights | | | | | | |
| --- | --- | --- | --- | --- | --- | --- | --- |
| Variable | Stigma (PSQ) | Openness to experience (CompactOE) | Valued actions (CompactVA) | Behavioral Awareness (CompactBA) | Depression (PHQ9) | Perceived Health (Quality of life;VAS) | Generalized Anxiety (GAD7) |
| Stigma (PSQ) | 0.000 | -0.142 | -0.062 | -0.022 | 0.087 | -0.022 | 0.000 |
| Openness to experience (CompactOE) | -0.142 | 0.000 | 0.000 | 0.450 | -0.010 | 0.000 | -0.218 |
| Valued actions (CompactVA) | -0.062 | 0.000 | 0.000 | -0.110 | 0.000 | 0.170 | -0.102 |
| Behavioral Awareness (CompactBA) | -0.022 | 0.450 | -0.110 | 0.000 | -0.060 | 0.055 | -0.177 |
| Depression (PHQ9) | 0.087 | -0.010 | 0.000 | -0.060 | 0.000 | -0.227 | 0.617 |
| Perceived Health (Quality of life;VAS) | -0.022 | 0.000 | 0.170 | 0.055 | -0.227 | 0.000 | -0.175 |
| Generalized Anxiety (GAD7) | 0.000 | -0.218 | -0.102 | -0.177 | 0.617 | -0.175 | 0.000 |

**Supplementary Material S3:** The case-dropping bootstrap simulation

To assess the centrality stability (how well the order of centralities are retained after observing only a subset of the data), we used the correlation stability coefficient, or for short CS-coefficient (quantification of stability). This coefficient indicates the percentage of cases that can be dropped, with a 95% probability of maintaining a .70 correlation compared to the complete data (Epskamp et al., 2018). The authors recommend values over .50 but no lower than .25.

**CS-coefficient**

Edge weight STABILITY: 0.59 [ the edge-weight accuracy is estimated when values are over .50 but not lower than .25]

- For more accuracy, run bootnet(..., caseMin = 0.514, caseMax = 0.676)

**
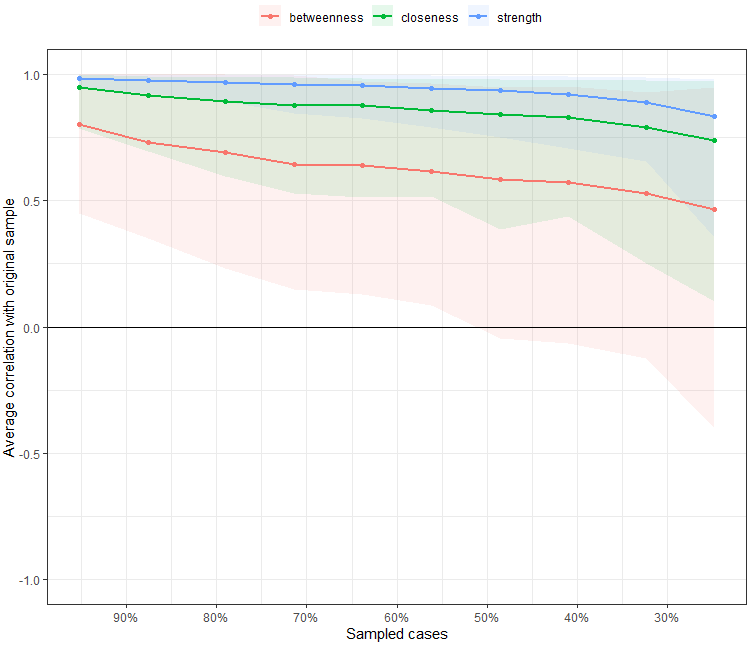
**

The stability plot after case-dropping bootstrap:

The stability of betweenness and closeness drop steeply while the stability of node strength is better. This stability has been quantified using the CS-coefficient, computed in R using the corStability function: corStability(boot2). The CS-coefficient indicates that betweenness (CS (cor = 0) = 0.05 and (CS (cor = 0.1) = 0.05 closeness are not stable under subsetting cases. Node strength performs better (CS (cor = 0.6) but does not reach the cut off of 0.5 from our simulation study required consider the metric stable. Therefore, we conclude that the order of node strength is interpretable with some care, with the order of the other two indices, betweenness and closeness are not.

**Edge weights CIs**

strength: 0.59

- For more accuracy, run bootnet(..., caseMin = 0.514, caseMax = 0.676)

We also estimated the edge weights confidence intervals (CIs; the CI equals the range of bootstrapped samples and is based on the two most extreme samples (minimum and maximum) to assess the precision with which PF processes were strongly interconnected within the network, with narrower CIs indicating better accuracy (Epskamp et al., 2018). The edge-weight bootstrapped CIs should not be interpreted as significance tests to zero, but only to show the accuracy of edge-weight estimates and to compare edges to one-another.

**
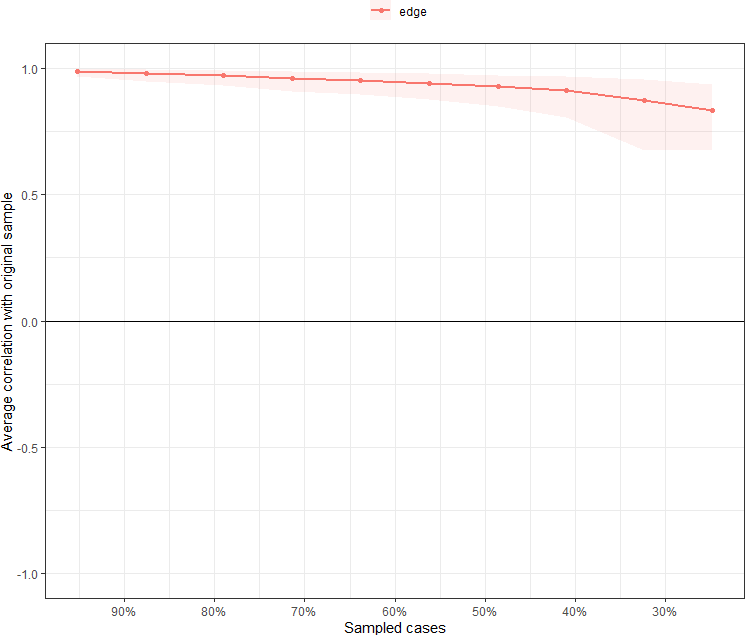
**

**Supplementary Material S4:** Participants’ list of skin diseases (diagnoses)

| **Primary Skin Diagnosis** | **N** |
| --- | --- |
| Psoriasis | 23 |
| Eczema | 16 |
| Alopecia | 11 |
| Acne | 7 |
| Actinic Keratoses | 7 |
| Lichen Sclerosis | 5 |
| Lichen Planus | 4 |
| Hydradenitis Supporativa | 3 |
| Keloid Scar | 3 |
| Lupus | 3 |
| Rosacea | 3 |
| Prurigo | 2 |
| Urticaria | 2 |
| Folliculitis | 2 |
| Chloasma | 1 |
| Cutaneous Vasculitis | 1 |
| Darier’s Disease | 1 |
| DRESS (Drug Reaction) | 1 |
| Erosive Pustular Dermatosis | 1 |
| Erythema Multiforme | 1 |
| Hyperhidrosis | 1 |
| Lichen Simplex Chronicus | 1 |
| Morphea | 1 |
| Palmoplantar Keratoderma | 1 |
| Polycystic Ovary Syndrome | 1 |
| Pressure Sore | 1 |
| Pruritis | 1 |
| Sweet’s Syndrome | 1 |
| Vitiligo | 1 |
